# GLP‐1 receptor agonists and serious adverse events: Evidence, uncertainty and clinical implications

**DOI:** 10.1111/joim.70170

**Published:** 2026-10-09

**Authors:** Peter Ueda

**Affiliations:** ^1^ Centre for Pharmacoepidemiology Division of Clinical Epidemiology Department of Medicine, Solna Karolinska Institutet Stockholm Sweden; ^2^ Centre for Diabetes Academic Specialist Centre, Region Stockholm Stockholm Sweden

**Keywords:** adverse effect, adverse outcomes, diabetes mellitus, drugs safety, obesity

## Abstract

Glucagon‐like peptide‐1 (GLP‐1) receptor agonists provide benefits in weight reduction, glycaemic control in Type 2 diabetes, and cardiovascular and renal outcomes. Their widespread use has given rise to concerns regarding rare but potentially serious adverse events. This narrative review appraises evidence regarding seven serious adverse events linked to GLP‐1 receptor agonists: thyroid cancer, non‐arteritic anterior ischaemic optic neuropathy (NAION), suicidal behaviours and self‐harm, diabetic retinopathy complications, acute pancreatitis, gallbladder and biliary disease and intestinal obstruction. Evidence from mechanistic studies, spontaneous reports, randomized trials and cohort studies is considered. Gallbladder and biliary disease has the most consistent evidence supporting a causal association. Worsening of diabetic retinopathy appears most consistent with early worsening after rapid glycaemic improvement in patients with pre‐existing retinopathy. For thyroid cancer, NAION, suicidal behaviour and self‐harm, acute pancreatitis and intestinal obstruction, evidence regarding causality remains uncertain. Rare events, incomplete ascertainment in trials and residual confounding in observational studies limit inference. Because these events are rare, even substantial relative risk increases generally correspond to small absolute risk increases in broad populations but may become relevant in patients with high baseline risk. Treatment decisions should consider the strength and uncertainty of safety evidence, individual baseline risk, expected benefits relative to available alternatives and patient preferences.

## Graphical abstract

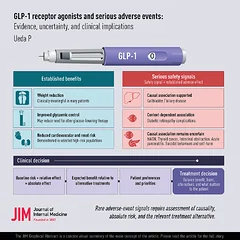

## Introduction

### GLP‐1 receptor agonists: mechanisms, clinical use and common adverse effects

Glucagon‐like peptide‐1 (GLP‐1) receptor agonists are incretin‐based therapies that enhance glucose‐dependent insulin secretion, delay gastric emptying and reduce appetite. Initially developed for the treatment of Type 2 diabetes, their use has expanded rapidly as newer agents produce substantial weight loss, and placebo‐controlled trials have shown reductions in cardiovascular events and kidney disease progression [1].

GLP‐1 receptor agonists and related incretin‐based therapies, including the dual glucose‐dependent insulinotropic polypeptide and GLP‐1 receptor agonist tirzepatide, have become among the most widely used drugs worldwide. Surveys indicate that approximately 5% of the national populations in the United Kingdom [2] and Finland [3] had used GLP‐1 receptor agonists by 2025. In Sweden, around 390,000 individuals had filled a prescription for a GLP‐1 receptor agonist in the same year, corresponding to approximately 5% of the adult population (www.socialstyrelsen.se/statistik‐och‐data/statistik/statistikdatabasen). National surveys from 99 countries suggest that more than one in four adults aged 25–64 years would be eligible for GLP‐1 receptor agonist treatment based on criteria combining obesity, Type 2 diabetes and related comorbidities [4].

Gastrointestinal symptoms, including nausea, vomiting, abdominal pain, diarrhoea and constipation, are the most common side effects of GLP‐1 receptor agonists, as repeatedly demonstrated in clinical trials [1]. Trials have also shown small average increases in resting heart rate [5], which may contribute to treatment discontinuation in some patients. In addition, rapid weight loss associated with GLP‐1 receptor agonist treatment could lead to functional consequences, including loss of muscle [6] and bone mass [7, 8], which could potentially be mitigated by adequate protein intake and progressive resistance exercise [7]. Weight loss may also necessitate adjustment of concomitant medications, including antihypertensive agents and glucose‐lowering therapies, to avoid hypotension and hypoglycaemia. Across key clinical trials of GLP‐1 receptor agonists [9, 10, 11, 12, 13, 14], 4%–16% of patients in the active treatment arm versus 3%–8% in the placebo arm have discontinued treatment due to treatment‐related adverse events.

For rare but serious adverse events potentially caused by GLP‐1 receptor agonists, the evidence is less robust. With millions of individuals worldwide receiving these drugs, understanding the evidence for such events and their implications for clinical decision‐making is important.

This review describes the study designs used to assess drug safety and reviews seven serious adverse events frequently discussed in relation to GLP‐1 receptor agonists (thyroid cancer and non‐arteritic anterior ischaemic optic neuropathy [NAION], suicidal behaviours and self‐harm, diabetic retinopathy complications, acute pancreatitis, gallbladder and biliary disease, and intestinal obstruction), assessing the evidence for causality. Clinical implications of the current evidence base are also discussed.

### Study types for assessing rare adverse events

Evidence for rare adverse drug events is derived from mechanistic studies, spontaneous reporting systems or case reports, randomized controlled trials and cohort studies.

#### Mechanistic studies

Mechanistic studies can provide information about whether a biologically plausible pathway exists through which GLP‐1 receptor agonists might cause a particular adverse event. For example, such studies may assess whether GLP‐1 receptors are expressed in tissues involved in the disease process. Because biologically plausible mechanisms do not necessarily translate into clinical effects, mechanistic studies are insufficient to establish causality [15].

Mechanistic evidence may be conceptualized as informing the prior probability of a causal association. Evidence from randomized and observational studies can subsequently modify that probability according to their estimates for effect on clinical outcomes. Similar clinical effect estimates may, therefore, warrant different interpretations depending on the strength of the underlying biological rationale.

#### Spontaneous reporting system data and case reports

Spontaneous reporting systems, such as the European Medicines Agency's EudraVigilance, are used to collect reports of suspected adverse drug reactions. Analyses of these databases, as well as case reports, rely on reporting from healthcare professionals, patients and manufacturers. Underreporting is substantial, the number of exposed patients is generally unknown, and reporting may be influenced by media attention and awareness of potential risks. Moreover, these data provide no or limited estimates for the counterfactual scenario—what would have happened to the patient had the drug not been used. Consequently, analyses of data from spontaneous reporting and case reports can be useful for signal detection but are insufficient for causal inference [16]. The difficulty of causal attribution at the individual level is illustrated by the Harmony Outcomes trial of albiglutide [17] in which five deaths were judged by investigators, who were masked to treatment allocation, to be treatment‐related; three of these occurred in patients randomized to placebo.

#### Randomized controlled trials

Randomized controlled trials provide the strongest evidence for causal effects because randomization minimizes confounding. However, most trials are designed to evaluate efficacy, and non‐prespecified safety outcomes may not be systematically captured. Post hoc safety analyses often rely on reports in trial databases, regulatory submissions or trial publications, which may contain discrepant information. This is illustrated by meta‐analyses with similar inclusion criteria that have reached different conclusions [18, 19, 20, 21] and by meta‐analyses reporting event counts that differ from those in the original trial publications [11, 20]. Further, reporting of non‐prespecified adverse events may be influenced by incomplete blinding. For GLP‐1 receptor agonists, effective blinding may be difficult to maintain because of their substantial effects on weight.

In addition, follow‐up is often short, and sample sizes are frequently insufficient to reliably assess rare adverse events or outcomes that emerge after prolonged treatment. Clinical trials may also exclude patients at increased risk of adverse events. For example, many GLP‐1 receptor agonist trials excluded individuals at high risk of suicide or history of pancreatitis through exclusion criteria or investigator discretion. As a result, assessment of adverse events in patient groups considered to be at particularly high risk is often not possible.

#### Cohort studies

Cohort studies using healthcare databases, such as the Scandinavian health registers [22, 23], complement clinical trials by including larger patient populations with longer follow‐up. This improves statistical power and allows for evaluation of safety in high‐risk subgroups that may be excluded in clinical trials.

Although propensity‐score‐based methods can adjust for a wide range of measured characteristics, residual confounding remains an important limitation. Further, data on drug adherence are not available, and low adherence may bias the findings towards the null. Moreover, given their increasing popularity, many of the comparator patients cross over to GLP‐1 receptor agonist treatment during follow‐up. Analyses censoring at crossover can introduce bias (which can be mitigated by inverse probability of censoring weighting) [24], and intention‐to‐treat exposure definitions attenuate potential drug effect estimates.

In addition, failure to use appropriate analytical frameworks, such as active‐comparator new‐user designs [25], may introduce time‐related biases and other design‐related sources of error [26, 27, 28]. The increased availability of healthcare databases and the intense interest in GLP‐1 receptor agonists have resulted in a rapidly expanding observational literature of variable methodological quality, complicating interpretation of the evidence base. For example, some studies have used exposure definitions which introduce immortal time bias and reported very large associations, including a 68% lower risk of complicated pancreatitis [29] and a 91% lower risk of all‐cause mortality after breast cancer diagnosis [30].

Taken together, each source of evidence has important limitations. For some outcomes, uncertainty regarding causality and potential effect magnitude may, therefore, persist despite more data being published. Uncertain estimates of potential causal effects may need to be approximated by combining evidence across study designs, and such judgements will inevitably depend in part on how the strengths and limitations of the individual studies are weighed.

## Evidence for rare but serious adverse events

### Thyroid cancer: from specific biological mechanism to broad safety signal (Table 1)

The concern regarding thyroid cancer emerged early during the development of GLP‐1 receptor agonists. Rodent studies demonstrated GLP‐1 receptor‐mediated thyroid C‐cell hyperplasia, C‐cell tumours (medullary thyroid carcinoma) and calcitonin release [31]. The clinical relevance of these findings was considered uncertain because GLP‐1 receptor expression is markedly lower, and the mechanisms for C‐cell activation differ for humans versus rodents [32]. Supporting this view, GLP‐1 receptor agonist treatment did not increase calcitonin levels in clinical trials [32].

Nevertheless, the rodent findings led to warnings regarding medullary thyroid carcinoma and, in the United States, contraindications for individuals with a personal or family history of medullary thyroid carcinoma or multiple endocrine neoplasia Type 2, a hereditary syndrome associated with an increased risk of this tumour type.

**Table 1:** Summary of evidence regarding glucagon‐like peptide‐1 (GLP‐1) receptor agonists and thyroid cancer.

Evidence domain	Summary of evidence
Mechanistic reasoning/evidence	Rodent studies demonstrated GLP‐1 receptor‐mediated thyroid C‐cell hyperplasia, medullary thyroid carcinoma and calcitonin release [31]. The relevance to humans is uncertain because GLP‐1 receptor expression and mechanisms of C‐cell activation differ between rodents and humans, and GLP‐1 receptor agonist treatment has not increased calcitonin concentrations in clinical trials [32]. The mechanistic rationale relates primarily to medullary thyroid carcinoma rather than thyroid cancer overall
Evidence from randomized controlled trials	Earlier meta‐analyses reported numerical imbalances in thyroid cancer events, although estimates were imprecise [42, 43]. A 2025 meta‐analysis reported an increased risk of thyroid cancer (OR 1.55, 95% CI 1.05–2.27) [18], and another recent meta‐analysis reported an OR of 1.37 (95% CI 0.82–2.31) [19]
Evidence from cohort studies	Some earlier cohort studies reported increased risks but used designs susceptible to time‐related biases [36, 37]. Larger studies using active‐comparator new‐user designs have generally not demonstrated an increased risk of thyroid cancer overall or medullary thyroid carcinoma specifically [38, 39, 40, 41]
Overall assessment	Human evidence does not consistently support a causal association. Some uncertainty remains because randomized estimates are inconsistent, but the original mechanistic concern relates primarily to medullary thyroid carcinoma, and larger contemporary cohort studies have generally been reassuring

Abbreviation: OD, odds ratio.

The original concern was specific to medullary thyroid carcinoma. However, medullary thyroid carcinoma is extremely rare (1%–2% of all thyroid cancers) [33]. In 2011, a study of spontaneous reporting system data evaluated thyroid cancer overall and found increased reporting for users of GLP‐1 receptor agonists [34]. As a result, much of the subsequent literature examined thyroid cancer as a composite outcome. The mechanistic rationale for this broadened outcome is less clear, although one small immunohistochemical study [35] reported GLP‐1 receptor expression in 3 of 17 human papillary thyroid carcinomas, the most common form of thyroid cancer.

Cohort studies subsequently indicated an increased risk of thyroid cancer associated with GLP‐1 receptor agonist use but used designs susceptible to time‐related biases [36, 37]. In contrast, larger cohort studies [38, 39, 40, 41] using active‐comparator new‐user designs have generally not demonstrated a significant association, neither for thyroid cancer overall nor for medullary thyroid carcinoma specifically.

Findings from randomized controlled trials have been less consistent. Meta‐analyses published during the late 2010s and early 2020s reported small numerical imbalances in thyroid cancer events, although confidence intervals were wide [42, 43]. In 2023, the European Medicines Agency concluded that the available evidence did not support a causal association between GLP‐1 receptor agonist use and thyroid cancer (https://www.ema.europa.eu/en/news/meeting‐highlights‐pharmacovigilance‐risk‐assessment‐committee‐prac‐23‐26‐october‐2023). An updated meta‐analysis [18], including trials published through November 2024, found a statistically significant increase in thyroid cancer risk among GLP‐1 receptor agonist users compared with controls (Mantel–Haenszel odds ratio [OR] 1.55, 95% CI 1.05–2.27) and the OR in another meta‐analysis, including trials through August 2025 was 1.37 (95% CI 0.82–2.31) [19].

Detection bias remains a possible explanation for some observed associations because thyroid cancer incidence is highly sensitive to the intensity of diagnostic investigation [44]. Whether GLP‐1 receptor agonist treatment materially increases diagnostic investigation or thyroid nodule detection through mechanisms such as increased medical attention during weight loss, as well as greater detectability of pre‐existing thyroid nodules following loss of adipose tissue, has not been established.

The evolution of this safety signal illustrates how a biologically specific concern can broaden as it moves from mechanistic studies to clinical and epidemiological research. What began as a concern regarding rodent C‐cell tumours gradually evolved into a broader question regarding thyroid cancer overall, with the scope of the safety question shaped in part by researchers’ choice of outcome definitions. Although large cohort studies using contemporary causal inference methods have generally not supported an association, the inconsistent findings from trial meta‐analyses indicate that some uncertainty remains.

### Non‐arteritic anterior ischaemic optic neuropathy: a safety signal arising from observational studies (Table 2)

Evidence regarding the potential association between GLP‐1 receptor agonists and NAION has been derived predominantly from observational studies. Interest in this outcome, a severe ophthalmologic event which can cause blindness, emerged following publication of a single‐centre study that reported more than a fourfold increased risk among patients with Type 2 diabetes and an almost eightfold increased risk among patients with obesity receiving semaglutide compared with other glucose‐lowering or weight‐loss medications [45]. However, the study included only patients referred to a specialized neuro‐ophthalmology centre and was susceptible to selection (collider) bias [46], which can result in associations that would not be observed in population‐based samples. As acknowledged by the authors [47], the data source and study design thus imposed important limitations on causal interpretation.

**Table 2:** Summary of evidence regarding glucagon‐like peptide‐1 (GLP‐1) receptor agonists and non‐arteritic anterior ischaemic optic neuropathy.

Evidence domain	Summary of evidence
Mechanistic reasoning/evidence	Rapid improvement in glycaemic control has been hypothesized to cause optic nerve swelling followed by ischaemia, analogous to early worsening of diabetic retinopathy [65]. The mechanism remains hypothetical
Evidence from randomized controlled trials	Randomized evidence is limited by very small numbers of events. In a meta‐analysis of 69 trials, 64 reported no events; across the remaining trials, 8 cases of ischaemic optic neuropathy occurred with GLP‐1 receptor agonists and 4 with comparators (OR 1.53, 95% CI 0.53–4.44) [64]. A separate pooled analysis identified 3 confirmed NAION cases with semaglutide or liraglutide and 5 with placebo [21]. Another meta‐analysis reported an increased risk with semaglutide (OR 3.92, 95% CI 1.02–15.02) [20], although discrepancies in reported event numbers limit confidence in this estimate
Evidence from cohort studies	The initial single‐centre study [45] reported large relative risk increases but was susceptible to selection bias. Subsequent cohort studies have produced heterogeneous findings. Studies using active comparators and more extensive adjustment have reported estimates ranging approximately from RR/HR 1.2 to 1.9 [58, 59, 60, 61, 62, 63], with some studies showing statistically significant increases and others no significant association or attenuation after more extensive confounder adjustment. Residual confounding remains an important concern
Overall assessment	A causal association remains uncertain. Several observational studies have reported increased risks, but estimates are heterogeneous and potentially affected by residual confounding. Randomized trials contain too few events to establish or exclude a moderate relative increase in risk

**^Abbreviations:^ :**NAION, non‐arteritic anterior ischaemic optic neuropathy; OR, odds ratio.

The publication of this study prompted numerous cohort investigations. However, several studies had design or reporting limitations, including prevalent‐user bias, immortal time bias, eligibility based on events occurring after start of follow‐up, selection bias, or suboptimal comparator groups, or had a methods description that was insufficient to determine how patients were selected, how exposures were defined, or the extent to which confounding had been addressed [48, 49, 50, 51, 52, 53, 54, 55]. The challenges associated with interpreting this literature are illustrated by several studies using the same underlying data source (TriNetX) that have reached different conclusions [50, 51, 52, 53, 56].

Studies using an active‐comparator new‐user design to compare GLP‐1 receptor agonists with SGLT2 inhibitors (an increasingly preferred comparator because of the partly overlapping indications of the two drug classes [57]) have reported mixed findings [58, 59, 60, 61, 62, 63]. Some have reported increased risks, whereas others have found no statistically significant associations or attenuation of associations after more extensive accounting for potential confounders. Across these analyses, hazard ratios or relative risk estimates have ranged from 1.23 (95% CI 0.65–2.33) [63] to 1.85 (95% CI 1.51–2.27) [59], although one study with limited confounder adjustment reported a substantially larger estimate (HR 6.35 [95% CI 2.88–14.75]) [58]. Importantly, confounding remains a concern because cardiometabolic risk factors are both indications for GLP‐1 receptor agonist treatment and risk factors for NAION, with studies differing substantially in the extent to which these factors, as well as history of NAION, were measured and adjusted for.

Evidence from randomized controlled trials is limited because NAION is extremely rare. In a meta‐analysis of 69 randomized trials of GLP‐1 receptor agonists, 64 reported no NAION events [64]. Across the remaining trials, eight cases of ischaemic optic neuropathy occurred among participants receiving GLP‐1 receptor agonists and four among those receiving comparator treatments (OR 1.53, 95% CI 0.53–4.44). Another pooled analysis of semaglutide and liraglutide trials, performed by Vilsbøll et al., identified three confirmed NAION cases among actively treated participants and five among placebo recipients, who had a nominally higher incidence [21]. A third meta‐analysis, which included only a subset of the semaglutide trials analysed by Vilsbøll et al., nevertheless found a larger number of events and a significantly increased risk (OR 3.92, 95% CI 1.02–15.02) for NAION [20]. This meta‐analysis also reported substantially different numbers of retinopathy events and risk estimates for the SUSTAIN‐6 trial than those reported in the original publication [11]. These discrepancies make it difficult to verify the analysis and limit confidence in its findings.

The most frequently proposed mechanism for the potential link between GLP‐1 receptor agonists and NAION relates to rapid improvement in glycaemic control [65]. Analogous to the early worsening of diabetic retinopathy observed following rapid correction of hyperglycaemia, large reductions in glycated haemoglobin have been hypothesized to induce optic nerve swelling followed by ischaemia. If correct, this mechanism would suggest that any increased risk may be concentrated among patients with diabetes experiencing substantial reductions in glycated haemoglobin and may not apply to individuals receiving GLP‐1 receptor agonists solely for weight management. It also raises the possibility that risk could vary across agents and doses.

Taken together, the available evidence remains difficult to interpret. The signal originated from a study with important methodological limitations, subsequent cohort studies have produced varying results, several of which were above the null, whereas others were compatible with no association, and randomized trials contain too few events to provide precise estimates. At present, the existence and magnitude of any causal association remain uncertain.

Across the available studies, NAION was rare, and absolute risk increases, if any, were small in most patients. Important unresolved clinical questions, therefore, concern the management of patients who develop NAION during GLP‐1 receptor agonist treatment and whether treatment should be avoided in individuals with a history of NAION, whose baseline risk of the disease in the contralateral eye is substantially elevated [66, 67]. Data addressing these questions are largely lacking, as patients with previous NAION have generally been excluded from studies, and the number of events in this group has been too small to permit meaningful analyses.

Accordingly, regulatory and professional organizations have reached different conclusions. The European Medicines Agency has recommended that the semaglutide product information is updated to include NAION as a very rare adverse effect and advises discontinuation of semaglutide if NAION is confirmed (https://www.ema.europa.eu/en/news/prac‐concludes‐eye‐condition‐naion‐very‐rare‐side‐effect‐semaglutide‐medicines‐ozempic‐rybelsus‐wegovy). The agency has not issued corresponding recommendations for other GLP‐1 receptor agonists. In contrast, the North American Neuro‐Ophthalmology Society and the American Academy of Ophthalmology [68] have emphasized the uncertainty of the evidence and the substantial cardiometabolic benefits of GLP‐1 receptor agonists, arguing that discontinuation may expose many patients to greater harms than the uncertain possibility of an increased risk for recurrent NAION.

### Suicidal behaviours and self‐harm: signals from case reports but not group‐level data (Table 3)

In July 2023, the European Medicines Agency initiated a review of a potential association between GLP‐1 receptor agonists and suicidal thoughts or self‐harm. The review was triggered by approximately 150 spontaneous reports submitted through pharmacovigilance systems (https://www.ema.europa.eu/en/news/ema‐statement‐ongoing‐review‐glp‐1‐receptor‐agonists).

**Table 3:** Summary of evidence regarding glucagon‐like peptide‐1 (GLP‐1) receptor agonists and suicidal behaviours and self‐harm.

Evidence domain	Summary of evidence
Mechanistic reasoning/evidence	GLP‐1 receptors are expressed throughout the central nervous system, and GLP‐1 receptor agonists can cross the blood–brain barrier [69, 70], providing biological plausibility for psychiatric effects. Increased risks of self‐harm have also been reported after bariatric surgery and with some weight‐loss medications [71, 72]. Conversely, neuroprotective effects of GLP‐1 receptor agonists have been proposed [73], and weight loss may have beneficial effects on psychological well‐being
Evidence from randomized controlled trials	Randomized trials and meta‐analyses have not demonstrated increased risks of suicidal behaviour or other psychiatric adverse outcomes [74, 75, 76]. However, events were rare, and individuals at high psychiatric risk were frequently excluded. A secondary analysis of tirzepatide trials showed no deterioration in PHQ‐9 scores [77]
Evidence from cohort studies	Large cohort studies using the active‐comparator new‐user design and evaluating suicide, self‐harm and related outcomes have not identified increased risks associated with GLP‐1 receptor agonist use [78, 79, 80]
Overall assessment	Available evidence does not support an increased risk of suicidal behaviour or self‐harm at the population level. However, suicidal thoughts may be incompletely captured, and evidence remains limited for patients at particularly high baseline psychiatric risk

A potential effect of GLP‐1 receptor agonists on suicidal behaviour was biologically plausible. GLP‐1 receptors are expressed throughout the central nervous system, and GLP‐1 receptor agonists can cross the blood‐brain barrier [69, 70]. In addition, previous studies have reported increased risks of suicide and self‐harm following bariatric surgery and during treatment with certain weight‐loss medications [71, 72]. Conversely, GLP‐1 receptor agonists have also been hypothesized to improve mental health outcomes. This hypothesis is supported by evidence suggesting overlapping biological mechanisms between depression and Type 2 diabetes, including neuroinflammatory pathways, as well as experimental findings indicating neuroprotective effects of GLP‐1 receptor agonists [73]. Moreover, weight loss in individuals with obesity may improve their psychological well‐being.

To date, randomized trials have not demonstrated increased risks of suicidal behaviour or other adverse psychiatric outcomes among individuals receiving GLP‐1 receptor agonists [74, 75, 76]. However, interpretation of these findings is complicated by the rarity of such events and by the exclusion of individuals at highest risk through trial eligibility criteria or investigator discretion. Consequently, statistical power has been limited. For example, a meta‐analysis of randomized trials published through August 2023 identified only 33 events of suicide, suicide attempt, suicidal ideation or self‐harm among 32,354 participants receiving GLP‐1 receptor agonists [74].

Evidence from newer trials has been similarly reassuring. A secondary analysis of tirzepatide trials found no deterioration in mental health status, assessed using the PHQ‐9 questionnaire [77]. Instead, scores improved slightly among participants receiving active treatment compared with placebo, although the magnitude of the difference was not considered clinically meaningful. Large cohort studies using the active‐comparator new‐user design evaluating suicide, self‐harm and related outcomes have likewise not identified increased risks associated with GLP‐1 receptor agonist use [78, 79, 80].

Overall, the available evidence does not support an effect of GLP‐1 receptor agonists on suicidal behaviour or self‐harm at the group level. This was also the conclusion reached by the US Food and Drug Administration (https://www.fda.gov/drugs/drug‐safety‐communications/update‐fdas‐ongoing‐evaluation‐reports‐suicidal‐thoughts‐or‐actions‐patients‐taking‐certain‐type) and the European Medicines Agency (https://www.ema.europa.eu/en/news/meeting‐highlights‐pharmacovigilance‐risk‐assessment‐committee‐prac‐8‐11‐april‐2024) following their reviews in 2024. Nevertheless, it should be noted that the original concern arose from reports of suicidal thoughts, an outcome that is difficult to measure and incompletely captured in both clinical trials and routine healthcare data.

### Diabetic retinopathy complications: the importance of the comparator and glycaemic change (Table 4)

The concern that GLP‐1 receptor agonists may increase the risk of diabetic retinopathy complications arose from the SUSTAIN‐6 trial [11], in which semaglutide was associated with a 76% relative increase in retinopathy complications compared with placebo among patients with Type 2 diabetes. Post hoc analyses suggested that the excess risk occurred early during treatment and was largely confined to patients with pre‐existing diabetic retinopathy who experienced substantial reductions in glycated haemoglobin during the first 16 weeks of treatment [81]. It was hypothesized that the observed increase reflected early worsening of existing retinopathy following rapid improvement in glycaemic control, a phenomenon previously described after initiation or intensification of insulin therapy [82, 83, 84].

**Table 4:** Summary of evidence regarding glucagon‐like peptide‐1 (GLP‐1) receptor agonists and diabetic retinopathy complications.

Evidence domain	Summary of evidence
Mechanistic reasoning/evidence	Rapid improvement in glycaemic control is an established cause of transient early worsening of diabetic retinopathy [82, 83, 84]. Post hoc analyses of SUSTAIN‐6 suggested that an excess risk was concentrated among patients with pre‐existing retinopathy who experienced substantial early reductions in HbA1c [81]. This supports a mechanism related to rapid glycaemic improvement rather than necessarily a direct retinal effect of GLP‐1 receptor agonism
Evidence from randomized controlled trials	SUSTAIN‐6 reported a 76% relative increase in diabetic retinopathy complications with semaglutide versus placebo [11]. No other randomized GLP‐1 receptor agonist trial has demonstrated an increased risk [12, 17, 85, 86, 87, 88, 89], although ascertainment and eligibility criteria have differed between trials. In a dedicated SURPASS‐CVOT retinopathy substudy, tirzepatide was not associated with increased risk compared with dulaglutide [92]
Evidence from cohort studies	Cohort studies assessing retinopathy outcomes for GLP‐1 receptor agonists versus comparators in patients with [93] or without history [94, 95] of diabetic retinopathy have not indicated increased risks
Overall assessment	The evidence is most consistent with a context‐dependent risk of early retinopathy worsening associated with rapid improvement in glycaemic control among patients with established retinopathy. A direct class‐specific retinal toxicity has not been established, and the observed association may depend on the comparator and magnitude and speed of glycaemic improvement

No other randomized controlled trial of a GLP‐1 receptor agonist has demonstrated an increased risk of retinopathy [12, 17, 85, 86, 87, 88, 89]. However, some trials excluded patients with proliferative retinopathy or diabetic macular oedema, did not include protocols for systematic retinal assessment and generally produced smaller reductions in glycated haemoglobin than those observed in SUSTAIN‐6. The ongoing placebo‐controlled trial dedicated to assess the effects of semaglutide on diabetic eye disease (NCT03811561) is expected to be completed in 2027.

If rapid improvement in glycaemic control is the primary mechanism, a similar signal might be anticipated with tirzepatide because of its substantial glucose‐lowering effect [90, 91]. However, the large cardiovascular outcomes trial of tirzepatide in Type 2 diabetes (SURPASS‐CVOT [10]) compared the drug with dulaglutide rather than placebo, as placebo‐controlled cardiovascular outcome trials were no longer considered ethically justified. Consequently, both treatment groups experienced improvements in glycaemic control, although the reduction was greater with tirzepatide. Accordingly, in a dedicated retinopathy substudy with systematic ophthalmologic assessment, tirzepatide was not associated with an increased risk compared with dulaglutide [92].

In addition, cohort studies assessing retinopathy outcomes for GLP‐1 receptor agonists versus comparators in patients with [93] or without history [94, 95] of diabetic retinopathy have not indicated increased risks.

In summary, whether GLP‐1 receptor agonists increase the risk of retinopathy complications may depend, at least in part, on the counterfactual (control scenario). If semaglutide is compared with placebo, rapid reductions in glycated haemoglobin may increase the risk of worsening retinopathy among patients with established retinopathy. In contrast, if semaglutide is compared with another treatment producing a similar improvement in glycaemic control, the excess risk may be absent.

Available clinical guidance generally indicates that this safety signal should not constitute a major obstacle to treatment, provided that appropriate retinal screening and follow‐up are performed [96, 97]. Early worsening of retinopathy is generally considered transient and is outweighed by the long‐term benefits of improved glycaemic control [96, 97].

### Acute pancreatitis: a longstanding concern with limited supporting evidence (Table 5)

Acute pancreatitis was one of the earliest adverse events linked to GLP‐1 receptor agonists. The initial concern was based on biological plausibility, as GLP‐1 receptor agonists may stimulate the exocrine pancreas to increase serum amylase and lipase concentrations [98]. In addition, numerous case reports described acute pancreatitis occurring after treatment initiation.

However, meta‐analyses of randomized controlled trials in both diabetes and obesity have not demonstrated an increased risk of pancreatitis, with relative risk estimates close to the null in many studies [98, 99, 100, 101, 102], although one meta‐analysis [103] published in 2025 reported an increased risk (relative risk 1.44, 95% CI 1.09–1.89) despite using broadly similar inclusion criteria as the previous ones. A cohort study using the active‐comparator new‐user design did not show any signal of increased risk versus SGLT2 inhibitor use [104].

**Table 5:** Summary of evidence regarding glucagon‐like peptide‐1 (GLP‐1) receptor agonists and acute pancreatitis.

Evidence domain	Summary of evidence
Mechanistic reasoning/evidence	GLP‐1 receptor agonists affect the exocrine pancreas and can increase amylase and lipase concentrations [98], and case reports have described pancreatitis following treatment initiation. Increased gallbladder disease could theoretically increase gallstone‐related pancreatitis. Conversely, reductions in triglyceride concentrations and alcohol consumption could theoretically reduce pancreatitis risk
Evidence from randomized controlled trials	Multiple meta‐analyses of randomized trials in diabetes and obesity have not demonstrated an increased risk of acute pancreatitis [98, 99, 100, 101, 102]. One meta‐analysis published in 2025 reported an increased risk despite broadly similar inclusion criteria [103]
Evidence from cohort studies	An active‐comparator cohort study comparing GLP‐1 receptor agonists with SGLT2 inhibitors found no increased risk of acute pancreatitis [104]
Overall assessment	Available comparative evidence does not support an increased overall risk of acute pancreatitis. A causal effect cannot be excluded, and evidence remains limited for patients with a previous history of pancreatitis

Assessment of drug‐induced pancreatitis is challenging. Diagnosis relies on a temporal relationship between drug exposure and disease onset, together with exclusion of alternative causes such as alcohol, gallstones and hypertriglyceridemia, although causation may be multifactorial. Hundreds of drugs have been implicated, but causality has been established in only a minority of them [105, 106].

It could be hypothesized that GLP‐1 receptor agonists affect pancreatitis risk in certain patients. In addition to potential direct effects on the pancreas, the increased risk of gallbladder disease [107] associated with GLP‐1 receptor agonists could secondarily increase the risk of gallstone‐related pancreatitis. Conversely, some GLP‐1 receptor agonists may reduce triglyceride levels [108] and alcohol consumption [109, 110], both of which could theoretically lower pancreatitis risk.

In summary, despite the absence of a consistent association in randomized trials and large cohort studies, acute pancreatitis remains an adverse event commonly attributed to GLP‐1 receptor agonists in the scientific literature, product information and public discourse. From a clinical perspective, the question is most relevant for patients with a history of pancreatitis or who develop pancreatitis during treatment. Data to guide management in these specific situations remain limited [111]. Many clinical trials excluded patients with history of chronic pancreatitis or acute pancreatitis. In the SELECT trial of semaglutide, 69 patients (both treatment groups combined) with a pancreatitis event more than 180 days prior to study start were included: None of them had an adjudicated acute pancreatitis event during follow‐up [112].

### Gallbladder and biliary disease: evidence suggestive of a causal association (Table 6)

An increased risk of gallbladder disease was observed in several early clinical trials of GLP‐1 receptor agonists. A 2022 meta‐analysis of 76 randomized controlled trials found that the risk of a composite outcome of gallbladder or biliary disease was significantly increased among participants randomized to GLP‐1 receptor agonists compared with placebo or comparator drugs (relative risk 1.37, 95% CI 1.23–1.52) [107]. Although increased risks were observed in both diabetes and weight‐loss trials, the associations tended to be stronger in weight‐loss trials. There was also evidence of greater risk with higher doses and longer treatment duration. Results were broadly consistent across key outcome components, including cholelithiasis, cholecystitis and biliary disease, as well as across individual GLP‐1 receptor agonists, except for oral semaglutide, for which no increased risk was observed. Subsequent meta‐analyses of tirzepatide trials [113, 114] and a 2025 meta‐analysis [115] reported largely similar findings. Large cohort studies have generally presented comparable findings [104, 116, 117].

**Table 6:** Summary of evidence regarding glucagon‐like peptide‐1 (GLP‐1) receptor agonists and gallbladder and biliary disease.

Evidence domain	Summary of evidence
Mechanistic reasoning/evidence	Rapid weight loss is an established risk factor for gallstone formation, potentially through increased biliary cholesterol saturation [118, 119, 120]. Experimental evidence also suggests effects of GLP‐1 receptor agonists on gallbladder motility and emptying and postprandial cholecystokinin secretion [121, 122, 123]
Evidence from randomized controlled trials	A meta‐analysis of 76 randomized trials found an increased risk of gallbladder or biliary disease with GLP‐1 receptor agonists (RR 1.37, 95% CI 1.23–1.52) [107], with stronger associations in weight‐loss trials and evidence of greater risk with higher doses and longer treatment duration. Findings were broadly consistent across cholelithiasis, cholecystitis, and biliary disease and across most individual agents. Subsequent meta‐analyses have reported similar findings [113, 114, 115]
Evidence from cohort studies	Large cohort studies have generally reported increased risks consistent with the randomized evidence [104, 116, 117].
Overall assessment	Evidence supports a causal association between GLP‐1 receptor agonist treatment and gallbladder and biliary disease. The association is supported by randomized and observational evidence and biologically plausible mechanisms

Several mechanisms have been proposed. Rapid weight loss has long been recognized as a risk factor for gallbladder disease [118, 119, 120] as mobilization of cholesterol from adipose tissue may increase biliary cholesterol saturation and promote gallstone formation. There is also experimental evidence pointing towards effects specific to GLP‐1 receptor agonists, including reduced gallbladder motility and emptying and suppressed postprandial cholecystokinin secretion [121, 122, 123].

In summary, among the outcomes reviewed, gallbladder and biliary disease has the most consistent evidence supporting a causal association with GLP‐1 receptor agonists. Increased risks have been observed consistently across randomized trials and cohort studies, and biologically plausible mechanisms have been proposed.

### Intestinal obstruction: mechanistically plausible but supported by limited evidence (Table 7)

Given that reduced gastrointestinal motility is a well‐established effect of GLP‐1 receptor agonists, a causal association with intestinal obstruction, including paralytic ileus, is mechanistically plausible [124]. Case reports describing intestinal obstruction among users of GLP‐1 receptor agonists have prompted regulatory action. The European Medicines Agency included intestinal obstruction as a potential adverse effect for liraglutide and exenatide, but not for other GLP‐1 receptor agonists, whereas the US Food and Drug Administration has listed ileus as a potential adverse event for all GLP‐1 receptor agonists, including tirzepatide (https://www.fda.gov/drugs/fda‐adverse‐event‐monitoring‐system‐aems/april‐june‐2022‐new‐safety‐information‐or‐potential‐signals‐serious‐risks‐identified‐fda‐adverse).

**Table 7:** Summary of evidence regarding glucagon‐like peptide‐1 (GLP‐1) receptor agonists and intestinal obstruction.

Evidence domain	Summary of evidence
Mechanistic reasoning/evidence	GLP‐1 receptor agonists reduce gastrointestinal motility, providing a biologically plausible mechanism for intestinal obstruction or paralytic ileus [124]. Case reports have contributed to regulatory warnings
Evidence from randomized controlled trials	Randomized evidence is sparse because intestinal obstruction is rare. One meta‐analysis of obesity trials reported no events [125], whereas another found no increased risk of paralytic ileus or other intestinal obstruction and no apparent imbalance in event numbers [115]
Evidence from cohort studies	Cohort studies have produced inconsistent findings. A UK study [126] reported HR 1.69 (95% CI 1.04–2.74) and a US study [127] HR 1.17 (95% CI 1.03–1.32) compared with SGLT2 inhibitors, whereas a Scandinavian study [128] found no increased risk (HR 0.83, 95% CI 0.69–1.01)
Overall assessment	Current evidence does not establish an increased risk of intestinal obstruction. A causal association is biologically plausible, but randomized evidence does not demonstrate a signal, and cohort studies have produced inconsistent estimates

Evidence from randomized trials remains limited because intestinal obstruction is rare. In a meta‐analysis of obesity trials, no events of intestinal obstruction were reported [125]. Another meta‐analysis found no increased risk of paralytic ileus or other types of intestinal obstruction and no apparent imbalance in event numbers [115].

Cohort studies with active‐comparator new‐user designs have evaluated the risk of intestinal obstruction among users of GLP‐1 receptor agonists compared to SGLT2 inhibitors in Type 2 diabetes. A UK study [126] and a US study [127] reported statistically significant risk increases (hazard ratios of 1.69, 95% CI 1.04–2.74 and 1.17, 95% CI 1.03–1.32, respectively), whereas a Scandinavian study [128] found no such association (hazard ratio 0.83; 95% CI 0.69–1.01).

Taken together, the available evidence does not provide convincing support for an increased risk of intestinal obstruction associated with GLP‐1 receptor agonists, although the link is mechanistically plausible.

## Discussion

Of the seven serious adverse events reviewed, only one (gallbladder and biliary disease) has consistent support for a causal association from both randomized controlled trials and cohort studies. For diabetic retinopathy complications, randomized evidence is most consistent with context‐dependent early worsening after rapid glycaemic improvement in patients with pre‐existing retinopathy. For the remaining outcomes, evidence is based on mechanistic hypotheses, case reports, cohort studies and meta‐analyses of randomized trials with few events, often with conflicting findings. Although the evidence for most of these safety signals remains uncertain, the potential risk increase may influence treatment decisions. The clinical relevance of these safety concerns depends on the possible absolute risk increase for the individual patient and how this compares with the expected benefits of treatment.

### Balancing clinical benefits and potential harms in individual patients

#### Baseline risks and absolute risks

Clinical decisions about GLP‐1 receptor agonist treatment require potential benefits to be weighed against potential harms. Benefits may include desirable treatment effects, such as weight loss or improved glycaemic control, as well as reductions in the risk of adverse outcomes such as cardiovascular or renal events. Harms may include increases in the risk of adverse events and treatment burden. For risk outcomes, the absolute effect depends on the baseline risk and the relative treatment effect. The overall benefit–harm balance also depends on the importance assigned to each outcome by the individual patient (Fig. 1). Although rarely quantified formally, this reasoning underlies clinical decision‐making and shared decision‐making.

**Fig. 1:**
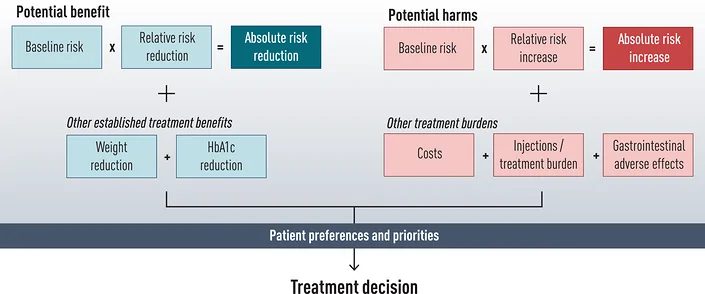
Conceptual framework for how baseline risks, relative risks, other treatment‐related benefits and harms and patient preferences are used to guide clinical decision‐making.

Fig. 2 summarizes absolute effects on weight and HbA1c and absolute risk reductions for cardiovascular events conferred by GLP‐1 receptor agonists. It also presents illustrative absolute risk increases for serious adverse events under the assumption that a causal association exists (using relative risks from studies reporting increased risks, i.e., a deliberately pessimistic scenario), both in broad patient populations and in selected high‐risk subpopulations.

**Fig. 2:**
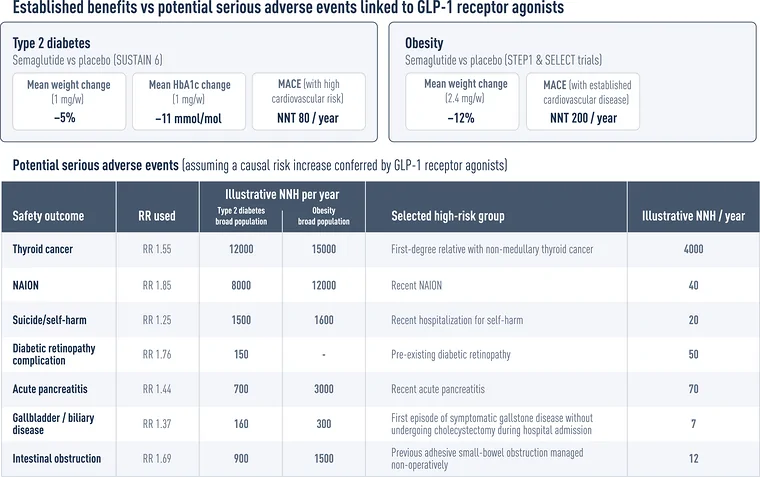
Established benefits of GLP‐1 receptor agonists and illustrative absolute risk increase under hypothetical causal associations. Input data and references are provided in Table S1 and Table S2. The number needed to harm (NNH) is estimated as 1/absolute risk increase per year. The absolute risk increase is estimated as baseline risk x (relative risk–1), assuming transferability of the relative risk across risk groups. Note that the NNH is per year and the NNH for longer periods can be roughly approximated by dividing the number with the duration of the period. The NNH are presented to put the safety concerns into a clinical context. Thus, the relative risks are selected from publications showing a potential increase conferred by GLP‐1 receptor agonists, although for most outcomes, the evidence regarding causality is uncertain. To avoid implying undue precision, NNH values were rounded to the nearest integer for NNH <20, to the nearest 10 for NNH 20–199, to the nearest 100 for NNH 200–1999 and to the nearest 1000 for NNH ≥2000. GLP‐1, glucagon‐like peptide‐1; NAION, non‐arteritic anterior ischaemic optic neuropathy.

Fig. 2 illustrates that the serious adverse events potentially associated with GLP‐1 receptor agonists are rare. Consequently, even if substantial relative risk increases would be assumed, they generally translate into small absolute risk increases in patients at low baseline risk. For example, given the low incidence of NAION among patients without a history of the condition, even a doubling of risk associated with GLP‐1 receptor agonists would correspond to a high number needed to harm. When baseline risk of potential adverse events is low, uncertain safety signals are unlikely to substantially influence treatment decisions (Fig. 2).

Safety concerns may become more important in patients at high baseline risk of the adverse event. For example, patients with previous NAION have a substantially increased risk of developing NAION in the contralateral eye. If GLP‐1 receptor agonists were to double this risk, the resulting absolute risk increase could be important. Thus, concerns regarding potential increases in the risk of serious adverse events with GLP‐1 receptor agonists are likely to be most relevant for patients with a high baseline risk of the event, such as those with a recent history of NAION, suicidal behaviour, or pancreatitis or symptomatic gallstone disease not treated with cholecystectomy (Fig. 2).

#### Expected benefits, alternative treatments and the importance assigned to outcomes

The balance is also affected by the expected benefits of treatment. When GLP‐1 receptor agonists are used primarily for cardiovascular risk reduction, for example, absolute benefits over the duration of clinical trials are relatively small, even in trials performed in patients at high cardiovascular risk and therefore most likely to benefit (Fig. 2).

The magnitude of expected benefit also depends on the treatment alternative. In trials such as SUSTAIN‐6 of semaglutide, active treatment was compared with placebo in patients with Type 2 diabetes and inadequate glycaemic control. Although investigators were instructed to intensify background glucose‐lowering therapy as needed, glycaemic control remained insufficient in the placebo group. Thus, the cardiovascular risk reduction from clinical trials is likely larger than the benefit of GLP‐1 receptor agonists compared with an alternative active glucose‐lowering strategy providing similar glycaemic control (although the cardiovascular risk reduction of GLP‐1 receptor agonists is likely also conferred by non‐glucose‐related mechanisms). This consideration is further reinforced by the availability of other treatment strategies for improving cardiorenal outcomes, including SGLT2 inhibition [129, 130], mineralocorticoid receptor antagonists [131] and potentially additional pharmacological LDL cholesterol lowering [132].

The relative importance of GLP‐1 receptor agonists versus other treatment alternatives may differ when weight reduction or glycaemic control is a major treatment goal. A substantial proportion of patients who tolerate treatment achieve clinically meaningful weight loss, which may improve quality of life, physical function and well‐being. Sustained substantial weight loss has historically been difficult to achieve with non‐surgical interventions. In patients with Type 2 diabetes, GLP‐1 receptor agonists can also produce substantial improvements in glycaemic control and may reduce the need for treatments associated with greater treatment burden and adverse effects, such as insulin. Thus, the balance between expected benefit and uncertainty regarding rare adverse events is likely to differ according to the indication for treatment and the availability and expected effects of alternative treatments.

Ultimately, however, the benefit–harm balance depends not only on the magnitude of absolute treatment effects, but also on the importance assigned to each beneficial and adverse outcome. Patients who place less value on weight reduction or other expected benefits of GLP‐1 receptor agonist treatment may, therefore, be less willing to accept potential increases in the risk of adverse events.

#### Heterogeneity of treatment effects

Finally, individual responses to a treatment may vary substantially [133]. It is conceivable, for example, that GLP‐1 receptor agonist treatment causes pancreatitis in some individuals through effects on gallbladder disease, whereas other patients avoid such an event through the drug's effects on alcohol consumption or triglyceride levels. Such heterogeneous treatment effects may help explain why individual case reports and group‐level data sometimes appear difficult to reconcile. Although such heterogeneous effects are speculative and difficult to predict without more granular data, they may nevertheless be relevant when interpreting safety signals in individual patients.

Taken together, the absolute risks, treatment effects and the relative importance of weight loss, glycaemic control, cardiovascular prevention and avoidance of potential serious adverse events will vary between patients, underscoring the role of shared decision‐making and individualized treatment decisions.

### Clinical consequences of safety concerns and the importance of adequately performed and interpreted research

Given their impact on treatment decisions and patient perceptions, drug safety concerns can have substantial consequences for individual patients. For example, patients may discontinue treatment due to such concerns [134], and physicians may be reluctant to prescribe GLP‐1 receptor agonists to patients with a history of NAION, pancreatitis, thyroid cancer or self‐harm, even when these patients stand to derive substantial benefits from treatment. Such decisions may partly reflect well‐described features of clinical decision‐making. Potential harms resulting from an active treatment decision may be weighted more heavily than harms resulting from withholding treatment, a phenomenon commonly described as omission bias [135].

In addition, adverse events occurring during treatment may be attributed to the drug even when its causal contribution is uncertain [136]. For example, even if treatment doubles the risk of an outcome, only half of the events among exposed individuals are attributable to the treatment. In practice, however, once a safety signal becomes established, subsequent events may readily be attributed to the treatment.

These considerations increase the importance of both adequately performed research and careful interpretation of that research. Mechanistic studies, case reports and spontaneous reporting systems are useful for signal detection but should not be interpreted as establishing causality. Observational studies should use designs that minimize avoidable sources of bias, including time‐related and selection biases, so that residual confounding remains the principal limitation rather than bias introduced by the analytical approach. Meta‐analyses of randomized controlled trials should carefully account for differences in data sources, inclusion criteria, outcome definitions and event ascertainment, and should report how these choices influence the findings. Across study designs, interpretation should emphasize effect estimates and their uncertainty rather than binary conclusions based on statistical significance.

### Future evidence on rare adverse events

Further research will improve the evidence on GLP‐1 receptor agonist safety, but some uncertainties are likely to persist. Larger randomized controlled trials may provide more precise estimates for serious adverse events, but for many safety concerns the most clinically relevant populations are those at high baseline risk, such as patients with previous pancreatitis, NAION or self‐harm (Fig. 2). Trials conducted specifically in these populations would provide the most directly applicable evidence but may be difficult to perform because of ethical and practical considerations.

The situation is further complicated by the evolution of clinical trials. As the benefits of GLP‐1 receptor agonists have become established, newer agents are increasingly evaluated against active comparators rather than placebo, particularly in Type 2 diabetes. For example, SURPASS‐CVOT compared tirzepatide with dulaglutide [10]. Although such designs are appropriate for evaluating comparative effectiveness, they do not provide information about the risks conferred by treatment versus no treatment. In the context of potential serious adverse events, however, this may be the causal contrast most relevant to clinical decision‐making. Safety evidence for newer agents may, therefore, become increasingly dependent on observational data and indirect comparisons.

In summary, among the serious adverse events reviewed, convincing evidence of an increased risk with GLP‐1 receptor agonists exists only for gallbladder and biliary disease; for diabetic retinopathy complications, the evidence is most consistent with context‐dependent early worsening in patients with pre‐existing retinopathy; for the remaining outcomes, the evidence is uncertain or conflicting. Translating these findings into sound clinical decision‐making requires consideration of the strengths and limitations of the underlying evidence, the magnitude and uncertainty of absolute risks, the expected benefits of treatment and individual patient preferences.

## Authors Contribution

**Peter Ueda**: Conceptualization; writing—original draft; investigation.

## Conflict of interest statement

The author declares no conflicts of interest.

## Funding information

Strategic Research Area Epidemiology programme, a Faculty Funded Career Position at Karolinska Institutet and grants from the Swedish Society of Medicine and ALF, Region Stockholm.

## Discloser

The funding sources had no role in any aspects of this narrative review.

## Use of artificial intelligence

ChatGPT (OpenAI; GPT‐5.5 and GPT‐5.6 Sol; accessed during manuscript preparation and revision, most recently 3 September 2026) was used to assist with language editing, including improving clarity, concision and structure; to suggest alternative wording and organization of manuscript sections; and to assist in developing the format of tables and figures. It was also used to assist with identification of references for baseline risks used in illustrative absolute‐risk estimates (Fig. 2); all these references were independently checked against the original sources by the author. AI‐generated or AI‐assisted text was independently checked, critically reviewed and revised by the author. All methodological judgements, evidence selection and interpretation, and conclusions were determined by the author, who takes full responsibility for the manuscript's content.

## Supporting information




**Table S1**: Input data for estimates used to approximate the number needed to treat (NNT) for cardiovascular events and number needed to harm (NNH) in a scenario with assumed causality between GLP‐1 receptor agonist use and serious adverse events.
**Table S2**: Input data for estimates used to approximate the number needed to harm (NNH) in selected high‐risk groups in a scenario with assumed causality between GLP‐1 receptor agonist use and serious adverse events.
