# Supplementary material for: GLP‐1 receptor agonists and serious adverse events: Evidence, uncertainty and clinical implications
Source: J Intern Med. 2026 Oct 9;300(6):758–78. doi: 10.1111/joim.70170 (PMC13741813; doi:10.1111/joim.70170)
Supplement: Supplementary file 1 [file JOIM-300-758-s001.pdf]

Supplementary Material

Ueda P. GLP-1 receptor agonists and serious adverse events: evidence, uncertainty, and clinical implications

Supplementary Table 1 Input data for estimates used to approximate the number needed to treat (NNT) for cardiovascular events and number needed to harm (NNH) in a scenario with assumed causality between GLP-1 receptor agonist use and serious adverse events.

Outcome	Relative risk used	T2D baseline rate (per 1,000 PY)	T2D absolute risk increase (%/year)	T2D NNH per year	Obesity baseline rate (per 1,000 PY)	Obesity absolute risk increase (%/year)	Obesity NNH per year	Note
**Major adverse cardiovascular event**	-	-	-	NNT 83	-	-	NNT 200	T2DM: In the SUSTAIN-6 trial of semaglutide vs placebo (Marso et al)[1] the incidence rate of MACE was 3.24 vs 4.44 events per 100 person-years, which approximates to 1.2% absolute risk reduction per year, giving an NNT of 83.<br><br>Effects on weight and HbA1c are taken from the clinical review report[2] providing dose-specific outcome data.<br><br>Obesity: In the SELECT trial of semaglutide vs placebo (as presented in the FDA review)[3] in patients with overweight or obesity and established cardiovascular disease but without diabetes, the incidence rates were 2.0 vs 2.5 events per 100 patient-years, giving an absolute rate difference of around 0.5% per year and an NNT of 200/year.<br><br>The % weight reduction vs placebo is taken from the STEP1 trial (Wilding et al)[4]
**Thyroid cancer**	1.55	0.146	0.008	12453	0.119	0.0065	15316	Relative risk: Positive meta-analysis point estimate (Silverii et al) [5]<br>Baseline rate T2DM: patients receiving DPP4 inhibitors in Scandinavia (Pasternak et al)[6]<br>Baseline rate obesity: estimate based on individuals with BMI>30 kg/m^2 in the NIH-AARP Diet and Health Study (Leitzmann et al.)[7]
**NAION**	1.85	0.143	0.0122	8227	0.102	0.0087	11545	Relative risk: Point estimate in observational study comparing GLP-1 receptor agonists vs SGLT2 inhibitors (Tesfaye et al.)[8]<br>Baseline rate T2DM: The estimated incidence rate in Olmsted County, Minnesota, US in individuals aged ≥50 y (Foster et al)[9] multiplied by 1.40 (the magnitude or increased risk among patients with diabetes according to Lee et al)[10]<br>Baseline rate obesity: Estimated in Olmsted County, Minnesota, US in individuals aged ≥50 y (Foster et al)[9]; estimate not specific for individuals with obesity.
**Suicidality (modelled as self-harm)**	1.25	2.700	0.0675	1481	2.500	0.0625	1600	Relative risk: Upper limit of 95% CI in observational study comparing GLP-1 receptor agonists vs DPP4 inhibitors (Shapiro et al)[11]<br>Baseline rate T2DM: Among individuals receiving DPP4 inhibitors in the study by Shapiro et al.[11]

								Baseline rate obesity: Individuals receiving intensive lifestyle treatment in Scandinavian Obesity Surgery Registry (Stenberg et al)[12]
**Diabetic retinopathy complications**	1.76	8.600	0.6536	153		-	-	Relative risk: Overall point estimate in SUSTAIN-6 of semaglutide vs placebo in type 2 diabetes and high cardiovascular risk patients (Marso et al).[1]<br>Baseline rate T2DM: Placebo group in SUSTAIN-6 (Marso et al)[1]
**Acute pancreatitis**	1.44	3.465	0.1525	656	0.822	0.0362	2765	Relative risk: Point estimate in positive meta-analysis (Wen et al)[13]<br>Baseline rate T2DM: Placebo group in SUSTAIN-6 (Sattar et al)[14]<br>Baseline rate obesity: Placebo group in the SELECT trial of patients with obesity and established cardiovascular disease (Kushner et al)[15]
**Gallbladder or biliary disease**	1.37	17.404	0.6440	155	8.500	0.3145	318	Relative risk: Point estimate in positive meta-analysis (He et al)[16]<br>Baseline rate T2DM: Placebo group in SUSTAIN-6 as presented in meta-analysis by He et al.<br>Baseline rate obesity: Placebo group in SELECT trial of patients with obesity and established cardiovascular disease. (Kushner et al)[15]
**Intestinal obstruction**	1.69	1.600	0.1104	906	0.993	0.0686	1459	Relative risk: Point estimate from positive UK observational study (Faillie et al)[17]<br>Baseline rate T2DM: Weighted incidence rate among SGLT2 inhibitor users in Scandinavian cohort study. (Ueda et al)[18]<br>Baseline rate obesity: Placebo group in SELECT trial of patients with obesity and established cardiovascular disease. (Kushner et al)[12]

Relative risks were taken from randomised trials or observational studies reporting increased risks (point estimates; for suicidality, the upper 95% confidence limit) to illustrate a scenario in which a causal association with GLP-1 receptor agonist use exists. Relative risks estimated in type 2 diabetes were also applied to individuals with obesity, although effects may differ between populations. For outcomes with high baseline rates, multiplying rates by relative risks slightly overstates one-year absolute risk differences.

Supplementary Table 2 Input data for estimates used to approximate the number needed to harm (NNH) in selected high risk groups in a scenario with assumed causality between GLP-1 receptor agonist use and serious adverse events.

Outcome	Relative risk used	High risk group	High risk group baseline rate (per 1000 PY)	High risk group absolute risk increase (%/year)	High risk group NNH per year	Estimation of the baseline risk
**Thyroid cancer**	1.55	First-degree relative with non-medullary thyroid cancer	0.4	0.02	4294	Incidence rate of thyroid cancer among patients receiving DPP4 inhibitors in Scandinavia (Pasternak et al)[6] multiplied by the incidence rate ratio of 2.9 estimated in study on the relative risk increase for family history of non-medullary thyroid cancer (Fallah et al)[19]
**NAION**	1.85	Recent NAION	29	2.5	41	Annualized risk estimated by taking the proportion of patients (14.7%) with NAION who had subsequent NAION in the fellow eye divided by the follow-up time (5.1 years) in a follow-up study of trial patients (Newman et al)[20]
**Suicidality (modelled as self-harm or suicide)**	1.25	Recent hospitalization for self-harm.	179	4.5	22	Estimates based on meta-analysis[21] of previous hospital-presenting self-harm/suicide attempt showing that 17.9% were rehospitalized for self-harm or committed suicide within from hospitalization.
**Diabetic retinopathy complications**	1.76	Pre-existing diabetic retinopathy	26	2.0	50	Patients in the placebo group with existing diabetic retinopathy in the SUSTAIN-6 trial (Vilsbøll et al)[22]
**Acute pancreatitis**	1.44	Recent acute pancreatitis	34	1.5	67	Annualized risk estimated by taking the proportion of patients (15.2%) with recent acute pancreatitis who experienced recurrent acute pancreatitis divided by the follow-up time (4.5 years) in a nationwide Danish study (Olesen et al)[23]
**Gallbladder or biliary disease**	1.37	First episode of symptomatic gallstone disease without undergoing cholecystectomy during hospital admission	370	13.7	7	Estimated using the relapse-free survival presented in the RELAPSTONE multicenter cohort study (Velamazan et al)[24]
**Intestinal obstruction**	1.69	Previous adhesive small-bowel obstruction managed non-operatively	120	8.3	12	Estimated using the 1-year readmission risk presented in the Bologna guidelines for diagnosis and management of adhesive small bowel obstruction. (ten Broek et al)[25] Reduced intestinal motility could theoretically act as a precipitating factor in obstruction caused by adhesion.

References

1. Marso SP, Bain SC, Consoli A, et al. Semaglutide and Cardiovascular Outcomes in Patients with Type 2 Diabetes. *New England Journal of Medicine*. 2016;375(19):1834-1844. doi:10.1056/NEJMoa1607141
2. Clinical Review Report: Semaglutide (Ozempic) - NCBI Bookshelf. Accessed September 26, 2026. https://www.ncbi.nlm.nih.gov/books/NBK544016/
3. FDA. Center for drug evaluation and research (215256Orig1s011). Published online 2024.
4. Wilding JPH, Batterham RL, Calanna S, et al. Once-Weekly Semaglutide in Adults with Overweight or Obesity. *New England Journal of Medicine*. 2021;384(11):989-1002. doi:10.1056/nejmoa2032183
5. Silverii GA, Marinelli C, Bettarini C, Del Vescovo GG, Monami M, Mannucci E. GLP-1 receptor agonists and the risk for cancer: A meta-analysis of randomized controlled trials. *Diabetes Obes Metab*. 2025;27(8):4454-4468. doi:10.1111/dom.16489
6. Pasternak B, Wintzell V, Hviid A, et al. Glucagon-like peptide 1 receptor agonist use and risk of thyroid cancer: Scandinavian cohort study. *BMJ*. 2024;385. doi:10.1136/BMJ-2023-078225
7. Leitzmann MF, Brenner A, Moore SC, et al. Prospective study of body mass index, physical activity and thyroid cancer. *Int J Cancer*. 2010;126(12):2947-2956. doi:10.1002/ijc.24913
8. Tesfaye H, Paik JM, Wexler DJ, et al. GLP-1RA and the risk of non-arteritic anterior ischaemic optic neuropathy in patients with type 2 diabetes: A population-based study. *Diabetes Obes Metab*. Published online October 17, 2025. doi:10.1111/dom.70200
9. Foster RC, Bhatti MT, Crum OM, Lesser ER, Hodge DO, Chen JJ. Reexamining the Incidence of Nonarteritic Anterior Ischemic Optic Neuropathy: A Rochester Epidemiology Project Study. *J Neuroophthalmol*. 2024;44(3):337-341. doi:10.1097/WNO.0000000000002102
10. Lee MS, Grossman D, Arnold AC, Sloan FA. Incidence of nonarteritic anterior ischemic optic neuropathy: increased risk among diabetic patients. *Ophthalmology*. 2011;118(5):959. doi:10.1016/J.OPHTHA.2011.01.054
11. Shapiro SB, Yin H, Yu OHY, Rej S, Suissa S, Azoulay L. Glucagon-like peptide-1 receptor agonists and risk of suicidality among patients with type 2 diabetes: active comparator, new user cohort study. *BMJ*. 2025;388. doi:10.1136/BMJ-2024-080679

12. Stenberg E, Bruze G, Sundström J, et al. Comparison of Sleeve Gastrectomy vs Intensive Lifestyle Modification in Patients With a BMI of 30 to Less Than 35. *JAMA Netw Open*. 2022;5(7):e2223927-e2223927. doi:10.1001/JAMANETWORKOPEN.2022.23927

13. Wen J, Nadora D, Bernstein E, et al. Evaluating the Rates of Pancreatitis and Pancreatic Cancer Among GLP-1 Receptor Agonists: A Systematic Review and Meta-Analysis of Randomised Controlled Trials. *Endocrinol Diabetes Metab*. 2025;8(5):e70113. doi:10.1002/edm2.70113

14. Sattar N, Lee MMY, Kristensen SL, et al. Cardiovascular, mortality, and kidney outcomes with GLP-1 receptor agonists in patients with type 2 diabetes: a systematic review and meta-analysis of randomised trials. *Lancet Diabetes Endocrinol*. 2021;9(10):653-662. doi:10.1016/S2213-8587(21)00203-5

15. Kushner RF, Ryan DH, Deanfield J, et al. Safety profile of semaglutide versus placebo in the SELECT study: a randomized controlled trial. *Obesity (Silver Spring)*. 2025;33(3):452-462. doi:10.1002/oby.24222

16. He L, Wang J, Ping F, et al. Association of Glucagon-Like Peptide-1 Receptor Agonist Use With Risk of Gallbladder and Biliary Diseases: A Systematic Review and Meta-analysis of Randomized Clinical Trials. *JAMA Intern Med*. 2022;182(5):513-519. doi:10.1001/JAMAINTERNMED.2022.0338

17. Faillie J, Yin H, Yu OHY, et al. Incretin-Based Drugs and Risk of Intestinal Obstruction Among Patients With Type 2 Diabetes. *Clin Pharmacol Ther*. 2022;111(1):272-282. doi:10.1002/cpt.2430

18. Ueda P, Wintzell V, Melbye M, et al. Use of DPP4 Inhibitors and GLP-1 Receptor Agonists and Risk of Intestinal Obstruction: Scandinavian Cohort Study. *Clinical Gastroenterology and Hepatology*. 2024;22(6):1226-1237.e14. doi:10.1016/J.CGH.2023.08.034

19. Fallah M, Pukkala E, Tryggvadottir L, et al. Risk of thyroid cancer in first-degree relatives of patients with non-medullary thyroid cancer by histology type and age at diagnosis: a joint study from five Nordic countries. *J Med Genet*. 2013;50(6):373-382. doi:10.1136/JMEDGENET-2012-101412

20. Newman NJ, Scherer R, Langenberg P, et al. The fellow eye in NAION: Report from the Ischemic Optic Neuropathy Decompression Trial Follow-up Study. *Am J Ophthalmol*. 2002;134(3):317-328. doi:10.1016/S0002-9394(02)01639-2

21. Carroll R, Metcalfe C, Gunnell D. Hospital Presenting Self-Harm and Risk of Fatal and Non-Fatal Repetition: Systematic Review and Meta-Analysis. *PLoS One*. 2014;9(2):e89944. doi:10.1371/JOURNAL.PONE.0089944

22. Vilsbøll T, Bain SC, Leiter LA, et al. Semaglutide, reduction in glycated haemoglobin and the risk of diabetic retinopathy. *Diabetes Obes Metab*. 2018;20(4):889-897. doi:10.1111/dom.13172

23. Olesen SS, Bruun NH, Davidsen L, et al. Natural History of Recurrent Acute Pancreatitis: Implications for Interventional Trial Design. *Aliment Pharmacol Ther*. 2026;64(7):976-984. doi:10.1111/apt.70820

24. Velamazán R, López-Guillén P, Martínez-Domínguez SJ, et al. Symptomatic gallstone disease: Recurrence patterns and risk factors for relapse after first admission, the RELAPSTONE study. *United European Gastroenterol J*. 2024;12(3):286. doi:10.1002/UEG2.12544

25. ten Broek RPG, Krielen P, Di Saverio S, et al. Bologna guidelines for diagnosis and management of adhesive small bowel obstruction (ASBO): 2017 update of the evidence-based guidelines from the world society of emergency surgery ASBO working group. *World J Emerg Surg*. 2018;13(1):24. doi:10.1186/S13017-018-0185-2
